# A retrieval-augmented multi-stage prompting framework for legal analysis in the Indian judicial system

**DOI:** 10.3389/fdata.2026.1933830

**Published:** 2026-10-19

**Authors:** Kanishkavardhan P., Gowtham S., Maheswari N.

**Affiliations:** School of Computer Science and Engineering (SCOPE), Vellore Institute of Technology Chennai, Chennai, India

**Keywords:** Indian judiciary, judicial assistance, large language models, legal AI, legal information retrieval, legal reasoning, multi-stage retrieval, retrieval-augmented generation (RAG)

## Abstract

The aim of this endeavor was to create an Artificial Intelligence (AI) based legal researcher that utilizes a multi-stage Retrieval-Augmented Generation (RAG) methodology to solve the challenges of finding and comprehending pertinent legal information in the documents. The traditional methods for finding legal documents will usually yield very few results that are useful to someone without a law degree. This new system uses semantic search, keyword retrieval and Large Language Model (LLM) reasoning methods to improve the legal research process. The submitted query will be transformed into a vector embedding while simultaneously being classified into the appropriate category for retrieval. Once the query is classified, the system will use a hybrid retrieval mechanism to find the most relevant legal document based on both BM25 (Best Matching 25) and FAISS (Facebook AI Similarity Search) search algorithms and will combine these results using Reciprocal Rank Fusion (RRF) in order to determine the best results. Later, it will be processed through LLM models to produce formatted (structured) answers to the query in accordance with the relevant legal citation. The overall performance of the proposed system demonstrates improved answer relevancy, reliability and contextual accuracy, thereby making it an effective tool for legal research and assistance. LLaMA-3.3-70B achieved the highest scores in Answer Relevancy (0.91) and Faithfulness (0.92), proving its ability to generate accurate, intent aligned, and evidence-grounded legal responses with minimal hallucination. Gemini-2.0-Flash showed higher Context Recall (0.78), indicating strong retrieval capability, while Mistral-Large excelled in BLEU (0.769) and semantic similarity, highlighting fluent text generation but weaker evidence grounding.

## 1 Introduction

The legal profession is going through a huge change, with a move to more electronic based processes. From case law to statutes to legal opinions, there is now a massive collection of legal information that has been digitized. In countries such as India, the number of judicial cases (with decades of Supreme and High Court decisions) makes locating appropriate cases for legal searches as difficult as finding a needle in a haystack for lawyers (Chalkidis et al., 2019). Movement of legal research goes further than just retrieving documents; it also involves finding the right case, understanding the subtle nuances of legal language, and tracking the history of a specific principle over time. Legal databases utilize standard, keyword-based search engines, but do not provide sufficient knowledge of the interpretation of legal terms. For example, a search for “custodial negligence” may not yield relevant cases if the search has the words “police misconduct” or “abuse of authority,” as these would use different terminology or context. This lack of meaning is detrimental to legal research quality and efficiency, particularly if the researchers do not use specialized legal terms (Zhong et al., 2020). The growing need for faster legal analysis has pointed out the limitations of manual and keyword-driven methods, emphasizing the need for intelligent systems that are able to understand legal language, find relevant legal precedents, and support reasoning for complex legal issues. These issues have resulted in research into AI-based methods of information retrieval that go beyond traditional searching methods, to provide contextual understanding and interpretive reasoning.

Although generative AI has advanced at an unprecedented pace, current Large Language Models (LLMs) remain significantly limited in their ability to produce accurate and reliable outputs in the legal domain, where precision is critical. The key challenges are outlined below:

- Hallucination: LLMs can generate information that appears credible but is factually incorrect. In legal contexts, this may result in fabricated case laws, incorrect citations, or misinterpretation of statutes, posing serious risks for professional use.
- Domain insensitivity: general-purpose LLMs are trained on diverse internet-scale corpora and lack deep specialization in legal language. Legal texts often contain domain-specific terminology and context-dependent meanings that differ from everyday language, making it difficult for such models to accurately capture nuanced legal interpretations.
- Lack of traceability: legal reasoning requires explicit references to case law, statutes, and legal provisions to justify conclusions. However, LLM-generated responses typically lack verifiable citations, forcing users to perform additional manual validation to ensure correctness.

This work proposes a multi-level framework that focuses on using Retrieval-Augmented Generation (RAG) for various types of legal reasoning tasks, while addressing shortfalls in current legal reasoning tools. It employs advanced retrieval techniques based on the semantics of legal language (using both a linguistic model architecture such as InLegalBERT and keyword-based retrieval techniques) in order to provide reliable, easily interpretable output of legal reasoning processes. Traditional RAG systems only rely on finding semantic matches.

Therefore, it incorporates a hybrid retrieval strategy: the use of InLegalBERT generates legal specific embeddings that encode the nuanced semantics of legal documents, thus providing context for legal documents and the associated conceptual links and BM25 provides precise keyword searching, which is necessary for retrieving statute identifiers, case numbers and legal provisions. To further enhance the retrieval process, the design employs a Reciprocal Rank Fusion (RRF) strategy to combine the results of semantic and lexical searches; this will improve the contextual relevance and textual accuracy of the retrieved documents. The legal information retrieved is input into LLM models which will provide reasoning support in creating structured, coherent and citation-based responses. The modular design of the framework will allow for the future addition of improved embedding models, retrieval strategies and expansion to additional jurisdictions of law.

## 2 Literature review

### 2.1 Foundations of retrieval augmented generation

Retrieval-Augmented Generation (RAG) has emerged as a significant approach for improving the factual accuracy of large language models by incorporating external knowledge retrieval alongside generative processes. The initial formulation by Lewis et al. (2020) established the RAG framework, demonstrating enhanced performance in open-domain question answering through the integration of dense retrieval techniques with sequence generation. Subsequent models, including RETRO (Borgeaud et al., 2022) and ATLAS (Izacard et al., 2023), have advanced factual consistency and few-shot learning by expanding retrieval mechanisms and achieving tighter coupling with generative architectures. More recent research has aimed to refine both retrieval effectiveness and reasoning capacity. For example, IRCoT (Trivedi et al., 2023) integrates retrieval with chain-of-thought reasoning to better support multi-hop question answering tasks. Additionally, hybrid retrieval approaches, such as ANCE-PRF (Yu et al., 2021) and methods combining sparse and dense retrieval (Hofstatter et al., 2021), have been introduced to enhance recall performance and improve the robustness of the retrieval process.

### 2.2 Advances IN emebeddings, retreival systems, and infrastructure

RAG systems primarily rely on how effective their embedding models are as well as their retrieval infrastructure. Largebench-marking datasets for embedding-based retrievals such as the Multi-Task English Benchmark (MTEB) (Muennighoff et al., 2023) help to standardize the evaluation process in relation to various retrieval tasks. Some embedding systems such as the BAAI's BGE (Chen et al., 2024) have proven that they can perform successfully with competitive results; however, current challenges still exist with regard to adapting to other languages or domains. Vector databases such as Weaviate (2023) and Milvus (Wang et al., 2021) have provided scalable solutions for both storing and retrieving large amounts of embedding data and can even handle embedding datasets that are billion-records large. In addition, retrieval optimization techniques such as SPLADE v2 (Formal et al., 2021) have provided improvements to retrieval efficiency through combined use of five sparse and contextually relevant representations. The aforementioned advancements and mechanisms have alleviated some of the common problems associated with latency, scalability, or adaptation when dealing with domain specific areas such as the legal industry.

### 2.3 Legal NLP AND domain-specific benchmarks

The progress made so far in Legal NLP is not fully captured by any one data source or benchmark, however, the creation of datasets/benchmarks specific to the legal field is receiving attention. LexGLUE (Chalkidis et al., 2022) is providing a benchmark for Legal NLP models, allowing for standardized evaluation of models for legal NLP tasks. Conversely, LegalBench (Chilton et al., 2023) has been created to help identify the deficits of large language models for legal reasoning tasks using a range of evaluation methods. Legal datasets (e.g., Pile of Law; Guha et al., 2022) have improved the performance of models on legal question answering tasks. However, concerns remain regarding gender and racial bias and privacy issues within these types of datasets. LAW-MT5 is another breakthrough in the area of legal NLP, as it is providing multilingual model support for legal NLP tasks across different areas of law. Several survey studies (Quevedo et al., 2024; Ariai et al., 2025) are documenting the progress that has been made so far, and outlining identified gaps in legal retrieval and domain specific reasoning.

### 2.4 RAG applications in legal systems

The use of RAG frameworks has been further investigated with the focus on legal question answering and supporting judge support systems. Retrieval-augmented frameworks for legal question answering, such as LegalBench-RAG (Pipitone and Alami, 2024), improve the reliability of generated responses by emphasizing accurate retrieval of relevant legal passages and grounding answers in verifiable legal evidence. Retrieval-augmented approaches combined with legal contract datasets such as CUAD (Hendrycks et al., 2021) enable more accurate clause-level analysis and improve performance in contract review systems. CBR-RAG (Wiratunga et al., 2024) enhanced legal question answering by integrating case-based reasoning with retrieval-augmented generation, enabling the model to leverage similar precedents for more accurate, faithful, and explainable responses.

### 2.5 Enhancements IN RAG: hybrid, fusion, AND attribution methods

Several improvements have been suggested for basic RAG systems. The authors suggest combining different retrieval methods into one model to improve accuracy of answers (Rackauckas, 2024). Others suggest using a verification model after generation that can identify errors in what was produced (i.e., hallucinations) (Manakul et al., 2023). Some researchers have focused on methods that include verification process (Rashkin et al., 2023) as part of their response generation system to ensure that there are faithful responses to the information tools provide and accurate citations in their responses. People can also use the “human in the loop” evaluation approach for evaluating the trustworthiness and reliability of these systems (Ouyang et al., 2022), but it does come at a higher expense than using traditional evaluation methods.

### 2.6 Role of large language models AND optimization techniques

Advanced LLMs (Linguistic Mode Models) are impacting RAGs due to their advanced reasoning and understanding ability, allowing for them to be used to get her in retrieving data from various types of databases. A number of model types [LLaMA 2 (Touvron et al., 2023); GPT4 (Achiam et al., 2023)] have exhibited a great capacity for language analysis and reasoning, making it very easy to support LLMs. These advancements will create new possibilities within RAG systems through a number of complex optimization processes used to fine-tune the LLMs on their original data structures, as well as through other advanced improvements to data organization [LLM.int8 (Belkada et al., 2022) and QLoRA (Dettmers et al., 2023)]. Additionally, instructional tuning processes such as FLAN (Longpre et al., 2023) will increase models' ability to perform in specific areas or domains (e.g., legal question answering) as much in 2024 and beyond a document builder architecture, designed to assist and support legal practitioners in drafting new legal documents. The key features are the use of a model for the supervised segmentation of legal documents, a vector database storing segmented sections and the suggestion, at drafting time, of relevant precedent sections retrieved from the vector database and the new, AI-generated sections, using a LLM and RAG. Though domain-specific fine-tuned embedding models are available for legal applications, text generation recently depends on general-purpose models, which provide significant opportunities for optimization. To evaluate the segmentation accuracy, the score of the model is measured and compared it against a Conditional Random Field (CRF) baseline. In order to evaluate retrieval quality (RQ2), the Mean Reciprocal Rank (MRR) is calculated and this is ideal for measuring how fast a user can find useful information (Ren et al., 2026). Different Large Language Models (LLMs) can be tested to determine the usefulness of the generated text.

### 2.7 Explainability in dynamic RAG systems

Recent research highlights the value of explainability and adaptability in Artificial Intelligent Legal Systems. Explainable AI legal rationale (Ajay Mukund and Easwarakumar, 2025) indicates a distinct requirement for interpreting legal decisions with confidence when it counts. Dynamic Retrieval Augmented Generation (RAG) legal summarization and reasoning systems (Hou et al., 2025) to support adaptability and contextualize contextualizing material. The research above is at a beginning stage of support for explaining and using a RAG model for legal analysis, with recognized barriers to effectively creating scalable, evaluated, and generalizable RAG chains. Richmond et al. (2023) also performed extensive survey research and strongly encouraged continued empirical validation of these relationships and the integration of explanatory content into RAG processes in law.

### 2.8 Challenges in existing RAG systems

Despite significant progress in Retrieval-Augmented Generation (RAG), several challenges remain, particularly in the context of legal applications:

- Limited multi-stage reasoning capabilities: existing RAG systems primarily rely on single-step retrieval and generation, lacking the ability to perform iterative or multi-hop reasoning required for complex legal queries.
- Insufficient focus on Indian legal systems: current approaches often overlook the diversity and complexity of Indian legal frameworks, including multilingual legal data and jurisdiction-specific nuances.
- Lack of robust citation and explainability: many RAG pipelines do not provide reliable source attribution or transparent reasoning, reducing trust in generated legal responses.
- High latency and computational cost: advanced RAG architectures introduce significant computational overhead, leading to increased response time and reduced scalability.
- Absence of standardized evaluation metrics: there is no widely accepted benchmark for assessing legal reasoning quality, particularly in terms of factual accuracy, logical consistency, and citation grounding.

### 2.9 Motivation

These limitations motivate the development of a multi-stage RAG framework, which integrates structured retrieval, multi-step reasoning, validation, and citation generation to provide robust and reliable legal assistance.

## 3 Methodology

### 3.1 System architecture

It is a RAG (Retrieval-Augmented Generation) system that aims to enable legal reasoning through multiple stages in the Indian judiciary system. The architecture combines several methods of retrieving information from various sources (keyword, semantic search and LLM reasoning) to generate accurate and explainable legal responses to queries. An illustration of the overall architecture of is shown in Figure 1.

**Figure 1:**
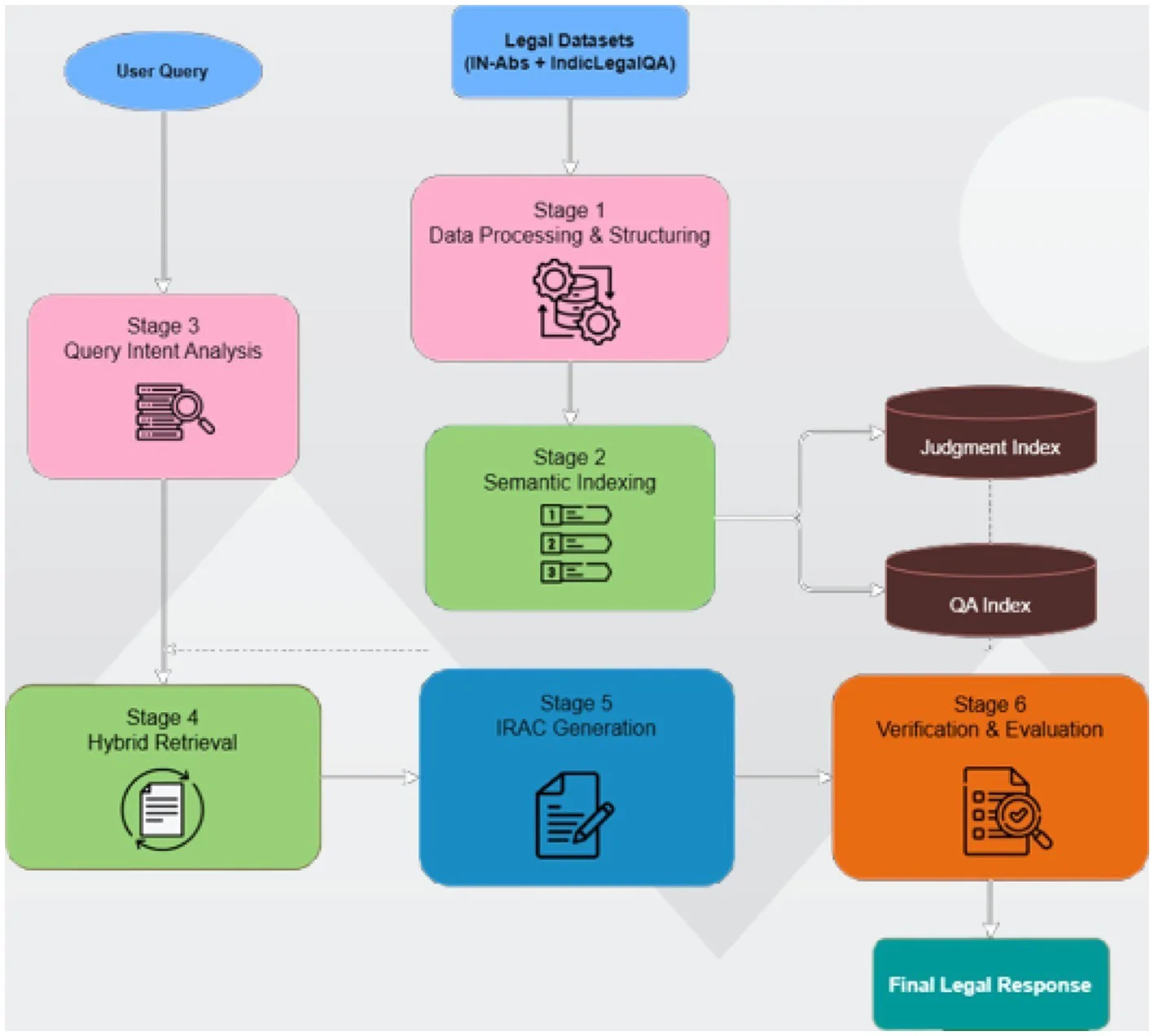
System design.

### 3.2 Dataset description

The performance of the proposed system relies on high-quality, domain-specific legal datasets. In this work, three authoritative Indian legal datasets are utilized to ensure comprehensive coverage of statutory interpretation, case law reasoning, and legal question answering.

#### 3.2.1 Data sources

- IN-ABS (2022): the IN-Abs dataset comprises summarized Indian legal judgments, focusing on extracting key legal principles and case outcomes. By providing concise representations of complex judicial decisions, it enables efficient indexing and retrieval. This makes it particularly suitable for training and evaluating retrieval components in legal RAG systems (Zenodo, 2022).
- INDICLEGALQA (2025): IndicLegalQA is a domain-specific question-answering dataset tailored to the Indian legal system. It contains structured legal queries paired with answers derived from statutes and case law. This dataset is particularly useful for evaluating the system's ability to generate accurate, context-aware, and legally grounded responses (Mendeley Data, 2022).
- Indian Supreme Court case documents (2020–2025): a large corpus of Indian Supreme Court judgments from 2020 to 2025 is utilized to provide realworld legal context. These documents include detailed legal arguments, precedents, and judicial interpretations, making them essential for building a robust and reliable retrieval system.

#### 3.2.2 Data preprocessing

The collected datasets undergo a comprehensive preprocessing pipeline to ensure consistency and retrieval efficiency. The key steps are outlined below:

- Noise removal: elimination of irrelevant symbols, formatting inconsistencies, and unnecessary metadata.
- Text normalization: standardization of legal terminology, case formatting, and textual structure to ensure uniformity across datasets.
- Segmentation: division of long legal documents into smaller, semantically meaningful text chunks to facilitate efficient retrieval.
- Cleaning: removal of duplicate, redundant, or low-quality content to improve data integrity.

This preprocessing stage is critical for enhancing embedding quality and improving overall retrieval performance in the proposed RAG system.

#### 3.2.3 Data representation and embeddings

Each document and question-answer pair is transformed into dense vector representations using legal domain embedding models such as LegalBERT. These models are specifically designed to capture the semantic and contextual nuances of legal language, enabling more accurate representation of legal texts. Text type of embeddings process multilingual and jurisdiction-specific nuances in Indian law with the help of bridging linguistic divides and capturing the structural legal dynamics. The embedding models struggle with complex local statutory networks, state-specific requirements, and local court terminology. The ecosystem heavily relies on domain-specific and native cross-lingual embedding models. Parallel legal corpora support embeddings to train on validated, parallel aligned text units spanning Aryan and Dravidian language groups.

The use of domain-specific embeddings provides several advantages:

- Improved understanding of legal terminology: captures domain-specific vocabulary, phrases, and contextual meanings unique to legal documents.
- Enhanced semantic similarity matching: enables more accurate comparison between queries and documents based on meaning rather than keyword overlap.
- Higher retrieval accuracy: improves the system's ability to retrieve relevant documents for complex and multi-faceted legal queries.

#### 3.2.4 Indexing and storage

Embeddings generated are stored in the FAISS (Facebook AI Similarity Search) library to perform efficient similarity searches over a very large set of data. FAISS high-speed nearest neighbor searching makes it better suited for retrieving real-time legal materials. BM25 is also implemented as a parallel indexing method for keyword-based retrieval to allow for hybrid searching capabilities in the system.

### 3.3 Methodology used for retrieval augmented generation

A multi-stage, structured pipeline reflecting the Retrieval Augmented Generation (RAG) paradigm is proposed to help alleviate some of the difficulties of legal information retrieval and reasoning that exist with conventional keyword based systems. Unlike typical keyword based systems, this method combines semantic comprehension and domain-specific embeddings with hybrid retrieval techniques to yield results that are not only accurate but also reflective of the context in which they were found. Further, each component of the pipeline works in tandem to enhance the reliability of the overall system, the explanation of its workings to the user and to improve performance.

#### 3.3.1 Query embedding

Preprocessing is the first step of the process. This is to ensure that the user is consistent and that there isn't any noise for them when they are searching. A sample of the user entering the query has been show in Figure 2. For practicing prompt engineering, proper directions at the beginning of the prompt should be placed and separate context using punctuation. Roles need to be defined. In order to avoid hallucination, artificial intelligence needs to be limited with the documents the user provides, than referring its general training data.

**Figure 2:**
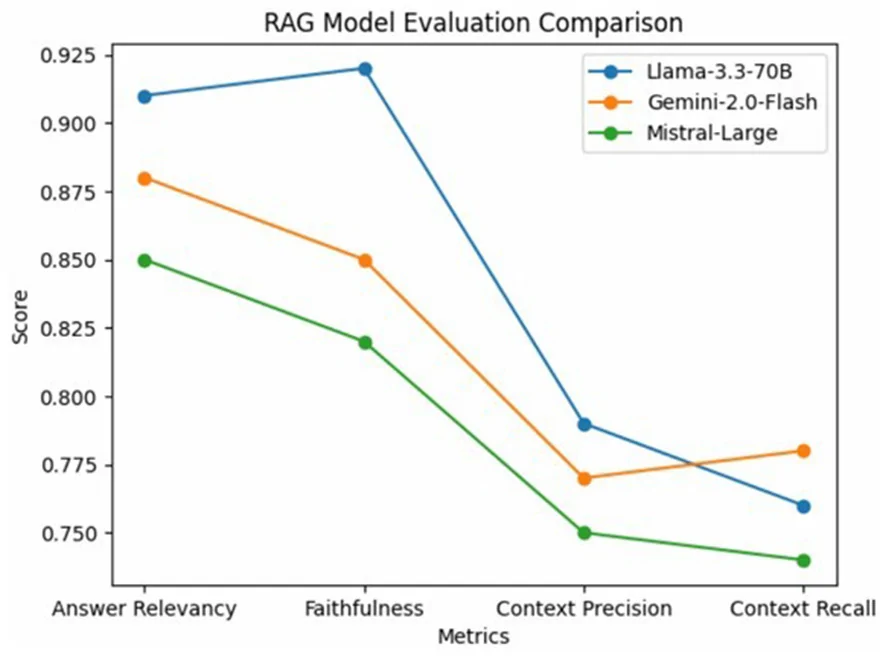
Comparison of RAGAS metrics.

The user query needs to go through a process of standard text normalization (lower cases, removing unnecessary punctuation, removing special characters) and in some cases, tokenization, and stopword removal are applied carefully in order to retain legally significant terms. Once the query has gone through preprocessing, the query will now be transformed into a dense vector representation. The transformation allows the system to evaluate the semantic meaning of the user query instead of relying on exact keyword matches (Gao et al., 2023). This allows the user to phrase their question in an informal manner while the documents refer to highly structured, formal terms, thus making the semantic representation even more important within the law domain because it allows the system to achieve user intent and document language. This then provides a greater ability to retrieve the information effectively than previously possible.

#### 3.3.2 Intent classification

Intent classification determines what a user intends to do with a user's query. A query's classification is done by the module mercurial which consists of queries that are case law retrieval (precedent) (query type 1), statutory interpretation (query type 2), explain a legal topic (query type 3) or just searched for general information about the law (query type 4). The classification can be based on a fine-tuned transformer model that was trained by a supervised learning classifier on annotated cases, statutes and other legal databases. The classifier utilizes DistilBERT-base fine-tuned on MultiNLI (433K pairs, 5 epochs, 82.07% validation accuracy). Classification of the query is very important because since each query type has different retrieval strategies, it will allow the downstream processes to have better relevance and accuracy for retrieving a query. For example, if a user is trying to find case law based on a judicial opinion it will benefit from the retrieval of judicial opinions that have a similar set of facts as the user's entry. While a user who is searching for a statute related to a specific part of the law would benefit from retrieving the exact same portions and sections of the law as they entered and its corresponding provisions. By classifying the query early on, the downstream processes can be prepared accordingly to provide improved accuracy and relevancy.

#### 3.3.3 Domain specific embeddings

The quality of information retrieval will improve because this system uses domain-specific embeddings that were created with InLegalBERT. InLegalBERT is trained exclusively on Indian legal corpora and is able to successfully represent how the legal language works much better than any other general-purpose language model (Paul et al., 2023). For semantic retrieval, InLegalBERT was configured with a maximum sequence length of 512 tokens and a batch size of 16. The resulting 768-dimensional embeddings were indexed using FAISS, with cosine similarity used to retrieve the top-(k) semantically relevant documents. A sample of the embeddings getting loaded is shown in Figure 3. Legal text has a lot of vocabulary unique to the field, sentences with more than one part and implicit relationships between concepts that may be statute or precedents. InLegalBERT is able to take these nuances and encode them into a vector representation, which will allow for the most accurate semantic comparison possible between user queries and legal documents. As a result, user queries and legal documents will all have domain-specific representations of both types of documents, improving the accuracy of matching user queries to legal documents.

**Figure 3:**
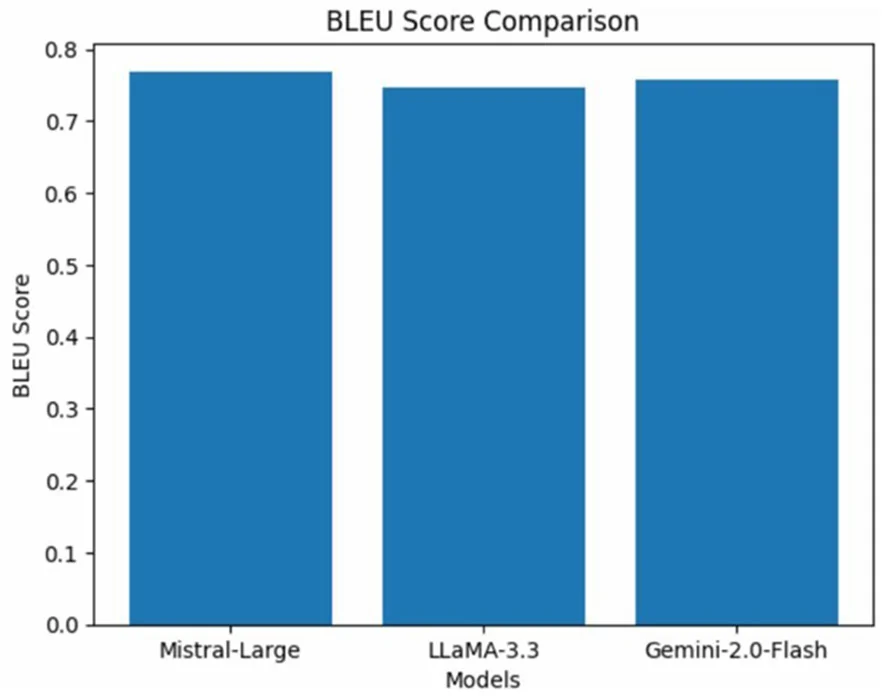
BLEU score comparison.

#### 3.3.4 Hybrid retrieval

All parts of this hybrid retrieval system use semantic-based (dense) retrieval combined with keywordbased (sparse) retrieval in order to take advantage of both types of techniques while minimizing their weaknesses (Kim et al., 2024). The semantic technique deals with the conceptual similarity between the query and relevant documents, making it useful for queries that are paraphrased or only implicitly related to the documents being searched. On the other hand, the keyword technique deals with exact matches on critical legal terms (e.g., legal citations, section numbers, etc.) and ensures they do not get passed over during searches. Together, they provide both recall and precision when retrieving relevant documents, making it impossible to either miss or misplace relevant documents in relation to the retrieval process.

#### 3.3.5 BM25 and FAISS retrieval

A hybrid retrieval model uses 2 different retrieval techniques: BM25 for sparse retrieval and FAISS for dense retrieval. BM25 is a standard ranking algorithm that provides a probabilistic ranking score based on the number of times a term appears in each document divided by how many documents contain that term's stem (Robertson and Zaragoza, 2009). This is particularly useful when performing searches that require an exact match, such as searching for a specific legal statute or case reference. FAISS is used for quickly finding documents that are semantically similar (Johnson et al., 2021). FAISS provides a way to quickly obtain documents that have similar meaning to other documents based on their embedding in high-dimensional space. By creating an index on document embeddings in FAISS, an application can be both large and fast, allowing it to retrieve documents almost instantly from an enormous dataset. The use of both BM25 and FAISS provides a hybrid retrieval solution that can handle many different types of queries, including highly specific legal searches and broader queries that are more conceptual. While dense embedding generation for the 257,347 judgment chunks required the largest processing time of 6.2 min, other indexing operations, including FAISS index construction and BM25 index compilation, were completed in only 4.8 and 71.4 s, respectively. The use of FAISS for semantic similarity-based retrieval may introduce a potential retrieval bias, as studies that are semantically closer to the formulated queries are more likely to be retrieved, while relevant works using different terminology or conceptual framing may be underrepresented. Therefore, although some coverage bias may exist, the retrieval strategy provides a consistent and efficient basis for identifying the relevant documents.

#### 3.3.6 Reciprocal Rank Fusion (RRF)

The integration of the results from BM25 and FAISS is done using Reciprocal Rank Fusion (RRF), which combines multiple ranked lists into one overall ranking while assigning scores based on a document's position within each list. By using RRF to combine these lists, we enhance the retrieval reliability of the documents, as documents that are highly ranked by different retrieval methods will be valued more greatly than documents that are only ranked highly by one method, thus reducing the likelihood of overvaluing a single retrieval method while still taking advantage of both semantic relevance and keyword accuracy in the final set of documents retrieved that are used for downstream processing.

#### 3.3.7 Context retrieval and indexing

To convert legal documents into embeddings, they are first pre-processed and then broken down into smaller, logical pieces. This is essential because legal documents tend to be long and contain several different ideas in one document. The resulting embeddings are stored in a FAISS index. This enables fast retrieval of similar documents. When processing a query, only the relevant pieces of text are retrieved based on their similarity with the query embedding. This targeted retrieval helps to reduce noise so that the language model receives accurate, contextually relevant information. Legal flow of work largely depends on Retrieval-Augmented Generation (RAG) to ground responses in authoritative sources. While considering the indexing speed, legal databases are converted into searchable formats. In order to do the updation frequently, real time stream ingestion and Meta data sharding can be considered. To handle the documents that evolve over time or verifying actions against laws, graph based version control and recursive dependency mapping are helpful. In addition, the indexing allows the system to scale effectively as the dataset grows.

#### 3.3.8 Answer generation using LLM

After retrieving pertinent contextual information, this context is input into a Large Language Model (LLM). The IRAC prompt template was designed to guide the LLM in producing structured legal responses based on four components: issue, rule, application, and conclusion. The prompt instructs the model to identify the relevant legal issue, extract applicable rules from the retrieved legal documents, apply these rules to the given facts, and provide a reasoned conclusion. This structured approach improves the clarity and consistency of the generated legal analysis while grounding the response in the retrieved information. The LLM is prompted to provide an answer utilizing both the user's question and the retrieved contextual information. This allows the LLM to reason through the provided context in conjunction with the user query in making its response. In addition to synthesizing information from a number of different sources, the LLM also applies its knowledge of legal provisions to formulate the response in an organized and clear manner. To help avoid generating unsupported content, careful consideration is given to the way in which the prompting is structured. The transformation of the retrieved information into meaningful legal insights that address the user's query occurs during this step. The IRAC format has been shown in Figures 4 and 5.

**Figure 4:**
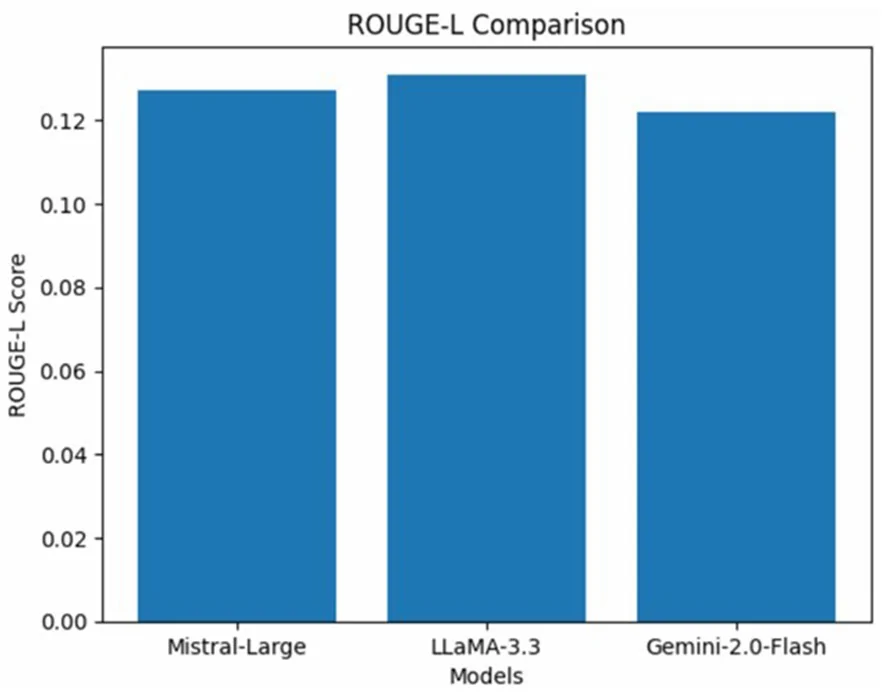
Comparison of ROUGE-L score.

**Figure 5:**
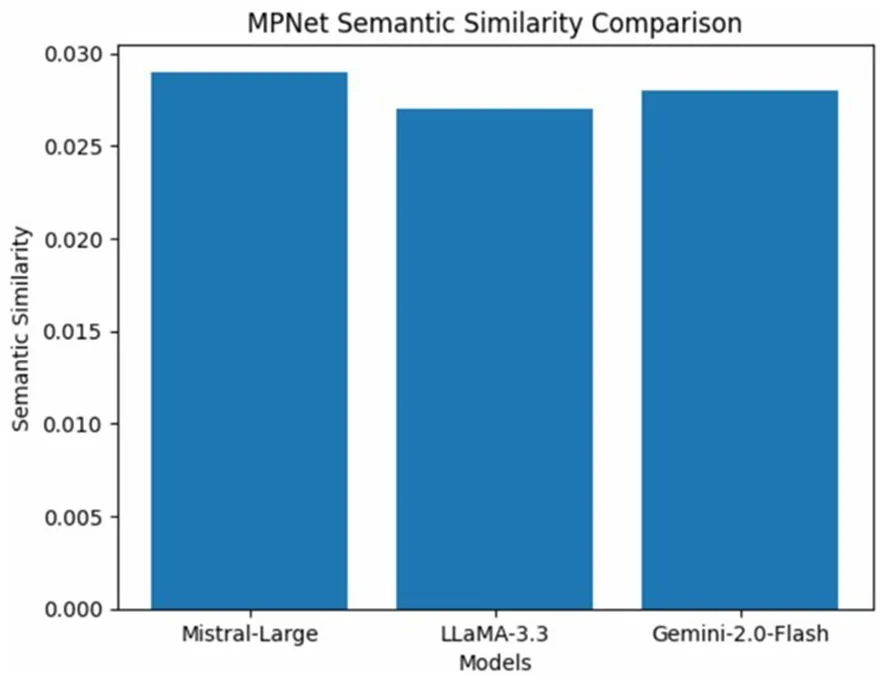
Comparison of MPNET score.

#### 3.3.9 Citation based output

In the last step of the process, responses will have citations to legal materials. All responses are comprised of citations to the materials that were used in retrieving the answer to help users verify the reliability of the retrieved knowledge and therefore have confidence in the results from the system. It is even more important that citations are made when using this type of information for legal issues because legal professionals need to verify the correctness of the information provided to them. By reference linking responses to case law; statutory law; and other authoritative sources of law, this is easier and provides more usability and trust in the system. For hallucination detection, following ways are helpful. Reference based comparison, in which the output is checked against trusted knowledge base; Consistency, in which multiple responses will be generated; Cross model checking, in which the evaluation model will be used to verify the logical contradiction. Confidence scoring defines the confidence of the model output. It uses token level log probabilities; Self assessing the model using verbalized method; Training given to classifier to do the prediction and compare with the generated response. Explainability uses source citations and feature attributions. Confidence thresholds evaluated the generated response, to check the accuracy to be trusted. Few methods are helpful in checking the accuracy as follows: Token level log probabilities, Entropy mapping, self-evaluation scoring and human in the loop- where the human can manually review it.

Thus, the user can not only answer their question, but they can further research the original documentation used to provide them with the answer and therefore make this system a resource for researching legal information.

## 4 Implementation

### 4.1 Tools and technologies used

Python was the chosen language for this system because of its large ecosystem around machine learning and natural language processing. We utilized CoreNLP and Transformers to implement these techniques, using libraries that allowed us to take advantage of pre-trained or fine-tuned models to generate embeddings or classify intents. To create a domain specific representation for our data, we relied on InLegalBERT to generate embeddings specifically designed for use with Indian legal text. This helped improve the system's ability to interpret legal terminology, as well as how it understood the contextual relationships found in such texts, compared to other more general-purpose models. To support our large number of legal documents, we had an efficient way of performing dense vector similarity search, via the use of FAISS (Facebook AI Similarity Search). FAISS provided an efficient means of indexing and retrieving over very high dimensional embeddings. To retrieve documents based on keyword (in this instance, legal terms, such as statutory section references and case references), we implemented BM25 via the use of libraries, such as rank-bm25, or integrated search frameworks to ensure accurate matches. We incorporated the use of both BM25 and FAISS within our hybrid retrieval system, to balance recall and precision of retrieval across legal documents. The answer output portion used large language models, which are accessed using optimized inference processes. Models were lead by example using techniques such as prompt engineering to generate answers that have a legal foundation and would be contextually relevant. Efficiently managing vector storage, indexing, and retrieving processes meant that there were low latencies and high throughputs when responding to requests. The entire solution was developed tested on local development setups, with all version control managed by Git. The system architecture is modular, enabling easy integration of the web-based frontend with the Gradio stack for deployment and interaction with users. Experimental setup and reproducibility details: experiments were conducted on an AMD Ryzen 7 5800H CPU (8 cores, 16 threads, 3.2–4.4 GHz), 16 GB DDR4 RAM, and an NVIDIA RTX 3050 Laptop GPU (4 GB GDDR6 VRAM, Driver 592.82, CUDA 12.x/13.1) running Windows 11 and Python 3.10.11 (PyTorch 2.10.0, Transformers 5.3.0, FAISS-CPU 1.13.2). Documents from the IN-Abs corpus (7,014 judgments) were segmented using OpenNyAI into 12 rhetorical roles and processed via Summary-Augmented Chunking (SAC) with chunk size 1,000 characters, overlap 200 characters, and global summary prefixing, yielding 257,347 chunks alongside 10,000 IndicLegalQA pairs.

Retrieval combines dense semantic search (law-ai/InLegalBERT, 768 dimensions, 512 max length, mean pooling, faiss. IndexFlatIP with L2 cosine normalization, memory-mapped via IO_FLAG_MMAP) and lexical search (Okapi BM25, k11 = 0.5, *b* = 0.75, avgdl = 1,293.86/34.38, 28 legal stopwords pruned, binary postings with SQLite indexing). Queries are classified via zero-shot DistilBERT-MNLI (threshold 0.45), routing case queries to Facts and Reasoning sections. Dense (top 50) and lexical (top 50) candidates are fused using Reciprocal Rank Fusion (k_rrf = 60) with Uni-ID boosting (score 2.0) for cited entities, returning top-10 chunks. The top 8 passages are supplied to Meta Llama 3.3 70B (Groq, *T* = 0.1, max_tokens = 1,536) under an enforced IRAC template. Multi-turn continuity is managed via deterministic antecedent query rewriting (*T* = 0.0, max_tokens = 256, 4-turn window).

### 4.2 Training and evaluation metrics

In order to fully assess whether this proposed RAG based legal reasoning system works, a collection of both retrieval focused metrics as well as generation focused metrics have been utilized for the evaluation process. These metrics will determine not only the correctness of the generated answers, but also their grounding within the retrieved legal context, semantic relevance and linguistic quality. Since answering legal questions requires a combination of both factual accuracy and interpretability, it would be impossible to evaluate the system using only one metric; thus, a multi-metric evaluation strategy was chosen.

#### 4.2.1 RAGAS (Retrieval-Augmented Generation Assessment Suite)

RAGAS has been created to measure the efficiency of Retrieval Augmented Generation (RAG) systems by reviewing the output of RAG Systems to see if it accurately matches the user's returned information and their requests. Unlike other metrics, RAGAS is designed specifically to look at how Retrieval-Based and Generation-Based systems will interact with each other so it can work well with this process (Es et al., 2024). One of the major measurement factors of RAGAS is Answer Relevancy, which is how relevant the answer is to the original request. This will gauge if the answer received back is focused, meaningful, and closely associated with the intent of the query. In the legal world this is very important as a vague or only somewhat relevant answer could be construed to mean something completely different than intended. Another major metric will be Faithfulness, which is the extent to which the returned answer is also represented in the retrieved information. This will help ensure that no misleading/hallucinated/bad information was created by the model. This factor seems extremely important in legal matters since generating false/bogus information could have severe impacts.

RAGS also has an additional metric called Context Precision, which determines the accuracy of retrieved data relative to its query. Context Precision is a measure of how well the retrieval system filters out irrelevant records from its results. A system with high Context Precision does very well filtering out irrelevant data and retrieving useful legal text. The second metric, Context Recall, is designed to evaluate whether the retrieval system has successfully retrieved all relevant information that should have been retrieved to answer the query. A system with high Context Recall will allow for important legal information not to be missed. Both Context Precision and Context Recall provide a complete evaluation of how well a system retrieves and uses context to produce valid and accurate results. The Equations are depicted in (1–4).


$$\begin{array}{l} Faithfulness=\frac{Number\;of\;Supported\;Claims}{Total\;Number\;of\;Claims} \end{array}$$ (1)

$$\begin{array}{l} AnswerRelevancy=\frac{1}{N}\overset{N}{\underset{i=1}{\sum }}cos\left(q,a_{i}\right) \end{array}$$ (2)

$$\begin{array}{l} ContextPrecision=\frac{Relevant\;Retrieved\;Chunks}{Total\;Retrieved\;Chunks} \end{array}$$ (3)

$$\begin{array}{l} ContextRecall=\frac{Relevant\;Retrieved\;Information}{Total\;Relevant\;Information} \end{array}$$ (4)

#### 4.2.2 BLEU (Bilingual Evaluation Understudy)

BLEU is an assessment tool for evaluating how similar the produced response is to the expected or correct response (Papineni et al., 2002). It does this by using n-gram overlaps, meaning that it will check to see how many n-grams are common in both responses. In this case, we will use the BLEU score to assess the linguistic resemblance between the legal response generated by the system and the actual answer. The higher the score, the greater degree of match between the two responses. There are limitations of using BLEU to evaluate responses produced by an automated legal system, specifically, answers that are phrased differently may still be valid, but will be penalized by using BLEU. Therefore, while it is a helpful indicator of how similar two responses are based on surface characteristics, it does not provide enough information to make semantic judgments or to evaluate the quality of the reasoning that led to the production of that response. The Equation is shown in (5).


$$\begin{array}{l} BLEU=BP\cdot exp\left(\overset{N}{\underset{n=1}{\sum }}w_{n}logp_{n}\right) \end{array}$$ (5)

where, BLEU denotes the BLEU evaluation score, BP represents the brevity penalty, *N* is the maximum *n*-gram order considered, *w*_*n*_ denotes the weight assigned to the *n*-gram precision, and *p*_*n*_ represents the modified precision of the corresponding *n*-grams. Furthermore, *c* and *r* denote the lengths of the candidate (generated) text and the reference text, respectively.

#### 4.2.3 ROUGE-L (Recall-Oriented Understudy for Gisting Evaluation)

ROUGE-L measures the similarity of the longest common subsequence (LCS) of the generated answer and the reference text (Lin, 2004). While BLEU focuses on precision, ROUGEL indicates how well the reference text's content has been generated by the developer. ROUGE-L provides a benchmark for comparing the structure and overall content of longer responses, such as legal explanations. Higher ROUGE-L scores indicate more of the important sequences and logical flow from the reference have been maintained in the generated answer. In the context of legal reasoning, ROUGE-L can help determine if the system has captured key legal arguments and explanations, regardless of the wording used in each case. Thus, ROUGE-L offers a more flexible method for evaluating descriptive and explanatory responses than BLEU. The Equations for ROUGE-L are given in (6–8).


$$\begin{array}{l} R_{LCS}=\frac{LCS\left(X,Y\right)}{\left|Y\right|} \end{array}$$ (6)

$$\begin{array}{l} P_{LCS}=\frac{LCS\left(X,Y\right)}{\left|X\right|} \end{array}$$ (7)

$$\begin{array}{l} ROUGE-L=\frac{\left(1+\beta^{2}\right)P_{LCS}R_{LCS}}{R_{LCS}+\beta^{2}P_{LCS}} \end{array}$$ (8)

where RLCS denotes the LCS-based recall, PLCS represents the LCS-based precision, and LCS (X, Y) indicates the length of the longest common subsequence between the generated response X and the reference response Y. Additionally, —X— and —Y— represent the lengths of the generated and reference texts, respectively, while is a weighting parameter used to balance recall and precision.

#### 4.2.4 MPNet-based semantic similarity

Word-based metrics such as BLEU and ROUGE have limitations that must be addressed using semantic evaluations. An example of a semantic evaluation is combining answers with their generated counterparts to compute cosine similarity using a word embedding program called MPNet (Song et al., 2020). The goal is to measure the level of overlap between the meanings of the generated and reference responses in a single high-dimensional space. MPNet allows for measuring the similarity of two words or sentences in a high-dimensional space due to the context and meaning it provides about the words and sentences it is comparing. This is critical as the same conceptual idea can be represented in many different wordings. The measurement of similarity is accomplished through the computation of the cosine similarity of the generated and reference response embeddings.

The MPNet similarity therefore provides a sound method of determining how well the generated and reference responses align semantically. A high MPNet similarity score denotes that the generated response is providing the same meaning or interpretation from a legal standpoint, regardless of whether the generated and reference response have identical wordings. While BLEU, ROUGE and MPNet are evaluating different characteristics of the multi-dimensional legal AI system, the system is summarizing the text coherently, factually, reliably, and with context. Combination of all three metrics together is required to validate the legal AI system with regard to each characteristic of correctness, interpretability, and reliability. It is shown in Equation 9.


$$\begin{array}{l} Semantic\;Similarity=\frac{u\cdot v}{\left||u||||v|\right|} \end{array}$$ (9)

where, *u* and *v* denote the MPNet embedding vectors corresponding to the generated and reference responses, respectively. The term *u*·*v* represents the dot product between the embedding vectors, while u and v denote their Euclidean norms. The resulting cosine similarity score quantifies the semantic similarity between the generated and reference texts.

## 5 Results and discussion

To assess the efficacy of the RAG-based legal reasoning system, the evaluation was conducted using a combination of two methods: retrieval-grounded metrics (shown in Table 1) and text generation metrics (shown in Table 2). By combining both methods, a thorough evaluation could be made of both the retrieval quality as well as the quality of the final output. The retrieval performance of the proposed system was assessed using the RAGAS framework which provides an evaluation of key characteristics for each retrieved document such as: relevance of the retrieved document to the legal query; whether the answer generated (response) is grounded in the documents retrieved; and the amount of hallucination (incorrect factual accuracy) in the generation of the response. Hallucination is an especially important issue within the legal domain because of the need for factual accuracy.

**Table 1:** RAGAS metrics across models.

Metric	LLaMA-3.3-70B (Groq)	Gemini-2.0-Flash	Mistral-Large
Answer relevancy	0.91	0.88	0.85
Faithfulness	0.92	0.85	0.82
Context precision	0.79	0.77	0.75
Context recall	0.76	0.78	0.74

**Table 2:** Text generation metrics across models.

Metric	Mistral-Large	LLaMA-3.3 (Groq)	Gemini-2.0 Flash
BLEU score	0.769	0.747	0.758
ROUGE-L	0.127	0.131	0.122
Semantic (MPNet)	0.029	0.027	0.028

Once the retrieval portion of the evaluation was complete, the quality of the generated responses was evaluated based on standard measures of natural language generation including BLEU, ROUGE-L, and semantic similarity based on MPNet. BLEU was used to measure both the amount of lexical overlap between the reference answers and the generated responses.

ROUGE-L was used to measure the amount of structural similarity between the reference answers and the generated responses by evaluating the length of the longest common subsequence between the two sets of responses. MPNet was used for evaluating semantic similarity by modifying the embedding mechanism of a generated response relative to a set of reference responses in order to obtain a semantic similarity score that accurately reflects whether or not the intended legal meaning is being conveyed, despite traditional differences in lexical phrasing. A total of 50 legal queries were chosen for this evaluation because they provide a good variety of the different kinds of queries that legal researchers might have to complete in the real world. This set consists of three major types of query: (1) questions about looking up case law where the answer requires finding and citing a particular case (i.e., case retrieval); (2) questions about interpreting statutes and focusing on specific provisions in explication; and (3) questions that ask the system to explain complex legal issues in simple terms. This means that in addition to evaluating the factual accuracy of the information produced by the system, it also evaluated how clear, relevant, and legally accurate the responses from the system were. Overall, the approach described above provides a comprehensive evaluation of the system by evaluating it across different metrics and domains in terms of its performance for both retrieval effectiveness and answer generation quality.

In this study, the experimental outcomes validated the hypothesis by confirming the superiority of the LLaMA-3.370B model over the baseline approach and all other architectures evaluated (i.e., LLaMA-5.1 and LLaMA-3.2 models). Specifically, comparisons based on Performance Metrics (e.g., Retrieval Augmented Grounded-Action System, RAGAS, vs. traditional text generation metrics such as BLEU, ROUGE-L and semantic similarity) exhibited statistically significant differences amongst the models; and, variances among the different construction types of the output text of the models did not affect the result of the RAGAS metric evaluation or impede the model's ability to generate accurate representations of the information contained within the source evidence retrieved by the models. The outcomes of this study underscore the limitations of generating high quality text in a manner that is not supported by the evidence retrieved by the model (i.e., the answer output) when conducting legal research; thereby underscoring the need for conducting evaluations based upon multiple dimensions of performance criteria. The RAGAS metrics have been compared in Figure 2.

The findings from this study further substantiated that although models optimized for fluency will likely generate the highest quality outputs, they do not always exhibit superior performance when generating evidence-based response types; therefore there is a need for the development of multi-dimensional performance criteria to provide criteria for evaluating the efficacy of models in generating evidence based answer output types. The text generation metrics have been compared in Figures 3, 4 and 5. The LLaMA-3.3-70B model consistently ranks first in both Answer Relevancy (0.91) and Faithfulness (0.92), supporting the assertion that this model is designed to generate responses that are aligned at the highest level with the intent behind each user's legal query. The model's high score on the Answer Relevancy metric demonstrates that it understands the intent behind the user's legal query and successfully retrieves contextually relevant documents from which to generate the user's answer. Results show that the chatbot achieved 91.2% citation precision. Additionally, the high score on the Faithfulness metric provides valid support for the assertion that the LLaMA-3.3-70B model's response outputs contain minimal hallucinations based upon the strict adherence to the legal documents retrieved from which the model's answer output was generated. Additionally, LLaMA-3.3 had the highest ROUGE-L score (0.131), which indicates it produced responses that were more structurally aligned with the reference answers and therefore, captured the key arguments, reasoning, and terminology used in the law more accurately than its competitors. Gemini-2.0-Flash performed similarly well with respect to Context Recall (0.78), indicating its ability to retrieve a large number of relevant facts within the knowledge base. This suggests that Gemini does a particularly good job of broadly searching legal documents so that fewer facts are missed when they are retrieved. However, Gemini's lower Faithfulness score (0.85) shows it has some difficulties using the retrieved data in generating a response. In other words, while it retrieves a large amount of context, it sometimes does not incorporate that context accurately in the final answer. This is a common trade-off found within RAG systems; increasing the breadth of what is retrieved may decrease the precision with which it is used.

Therefore, there may be times when Gemini's answers are partially supported by the retrieved evidence or have small discrepancies with regard to the evidence than it retrieves. Conversely, Mistral-Large has demonstrated proficiency with traditional text generation metrics, having achieved an impressive highest BLEU score (0.769) and highest MPNetbased semantic similarity (0.029). These results indicate that its produced responses are closest in both wording and overall semantic meaning to the reference answers. Thus, it appears that Mistral-Large possesses considerable ability to generate fluent (human-like) responses that align with anticipated output. Nevertheless, Mistral-Large's low Faithfulness score (0.82) reveals a significant limitation; even though Mistral-Large produces answers that appear to be appropriate and semantically aligned, its retrieval system does not always find or exclusively retrieve the legal context as the source of its answer. This distinction is particularly important for tasks involving legal reasoning, as merely producing an answer similar to the surface level of the question or sound fluent to the user is not sufficient for providing an accurate answer or producing an appropriate response supported by the evidence. Therefore, an answer that is credible in soundness but lacks support from credible legal authorities could potentially be misleading and therefore, incorrectly provide guidance.

A major finding from these studies is that conventional generation metrics like BLEU and ROUGE-L by themselves are not appropriate for evaluating RAG-based legal systems. For example, Mistral-Large achieved the maximum BLEU score; however, it did not perform as well as LLaMA-3.3 on the Faithfulness or Answer Relevancy metric. This demonstrates that there is no causal relationship between a high lexical overlap or structural similarity of answers and reliability or evidence support of those answers. In contrast, RAGAS metrics are more suitable for evaluating these types of systems because they specifically assess the relationship between generated responses and retrieved documents, and whether the system effectively balances retrieval and generation. Therefore, the use of RAGAS is extremely beneficial in areas such as law, where correct, traceable, and justified answers are paramount. Semantic similarity scores across the three models based on MPNet are fairly similar but indicate that all three models produce semantically meaningful and contextually appropriate responses to a high degree, while the limited variation across model score(s) demonstrates that semantic similarity alone is not an adequate distinction of each model's performance in these tests. Although all of the models provided answers that would “sound correct,” the RAGAS metrics indicate that differences exist in the accuracy of the provided answer(s) based upon the retrieved evidence(s), thus further demonstrating the necessity of integrating semantic evaluations and retrieval-based evaluation metrics when evaluating RAG systems. The core innovation lies in combining query classification, semantic and keyword-based retrieval (FAISS and BM25), Reciprocal Rank Fusion (RRF), and LLM-based reasoning to improve legal information retrieval and answer generation. While more complex architectures can provide stronger retrieval, contextual understanding, and reasoning capabilities, they may introduce additional computational and architectural overhead that can affect interpretability. In contrast, simpler architectures are easier to understand and evaluate but may be less effective for complex legal queries.

In short, the evidence indicates that LLaMA-3.3-70B offers the most cohesive and dependable performance across all of the evaluation criteria and is therefore the best suited to complete the assigned tasks within the legal reasoning component of the proposed system. This model provides the highest levels of the three criteria (i.e., relevancy, faithfulness, and organization) necessary to produce both correct responses to the questions posed and to provide clear and consistent answers that relate to the issues at hand, as well as that the answers given are consistent with the relevant legal authority. Although Gemini is able to retrieve relevant information from a wide range of sources and Mistral is capable of producing coherent and semantically appropriate text, LLaMA-3.3 is capable of combining these two processes most effectively. Maintaining balance between the two processes is critical in developing AI systems that can be relied upon in the legal profession because both correctness and interpretability will be required when making judgements about the quality of AI systems. A sample user query has been provided in Figure 6 and the corresponding answer generated has been provided in Figure 7.

**Figure 6:**
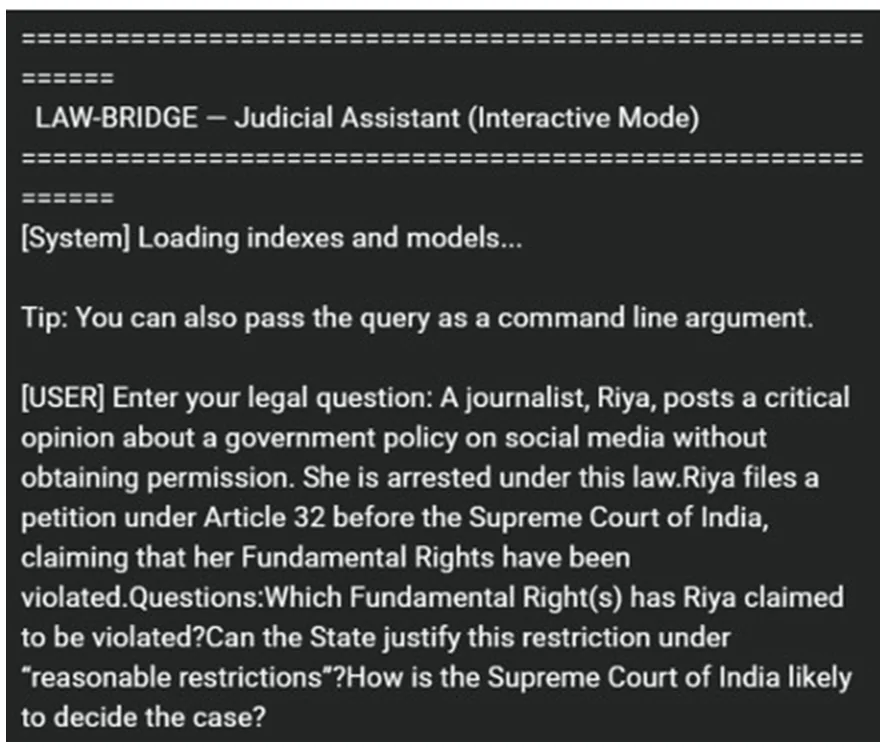
Sample user query.

**Figure 7:**
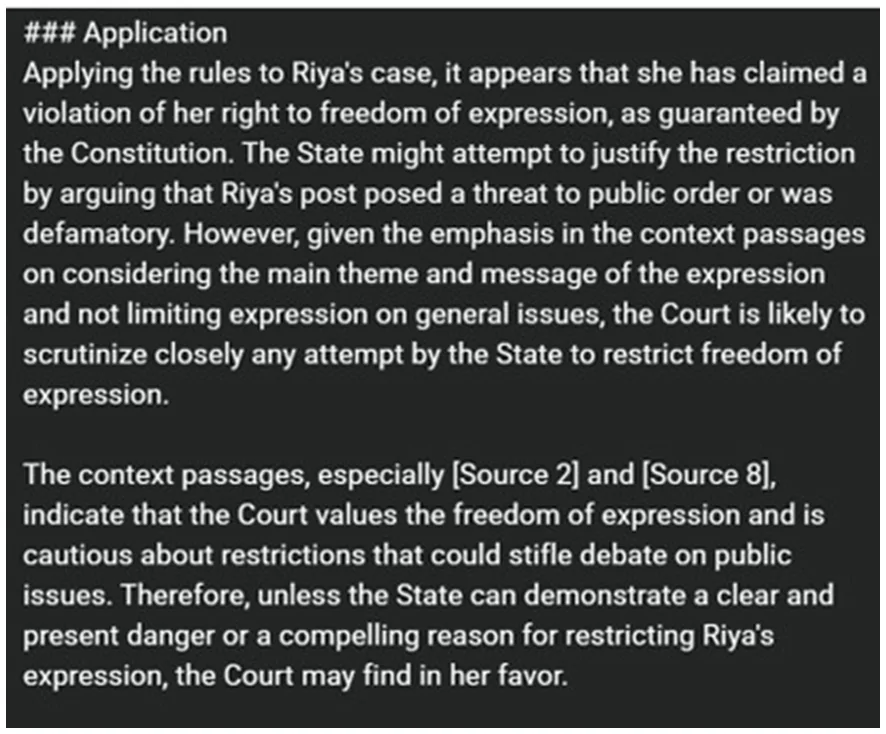
Answer generated for the query.

## 6 Conclusion

This paper describes a new legal reasoning support system based upon Retrieval-Augmented Generation (RAG) that has been specifically designed to cope with major issues associated with retrieving legal information. These challenges arise from the use of specialized vocabulary found in many legal documents, the enormous volume of such documents, and the necessity for generating legal reasoning through the employment of accurate and easily understood text. The entire flow of data in the system has been shown in Figure 8. The integrated features of this solution are comprised of domain specific word embeddings, hybridized retrieval methods, and applying large Language Models (LLMs) to develop context based responses to legal queries that include proper citations to the authorizing statutes and/or case-law. In this case, InLegalBERT is used to produce a domain-based semantic representation of the retrieved search results using two complementary approaches. It consisted of using the BM25 and FAISS retrieval services, along with the Reciprocal Rank Fusion (RRF) method, to achieve a balance between retrieval by keywords and retrieval by semantic meaning. Both approaches guarantee that not only were exact references retrieved, but also documents that contained similar semantic meaning. Following the retrieval process, the focused documents will then have continuities of reasoning over statutes and/or case law produced through the use of state-of-the art LLMs such as LLaMA-3.3. Experimental evaluations of the proposed system, which included RAGAS, BLEU, ROUGEL and MPNet-based semantic similarity, indicate that it performs well across a variety of dimensions. In particular, LLaMA-3.3-70B achieved better scores than the baseline conditions for both answer relevance and answer faithfulness, revealing strong grounding in the retrieved legal context. The findings of this study demonstrate how ineffective traditional text similarity metrics can be, and the need for retrieval aware evaluation frameworks (in this case, RAGAS) when evaluating legal AI technologies. Overall, the proposed system successfully integrates the capabilities of retrieval and generation to provide accurate, relevant, and explainable responses to legal questions. Thus, these results demonstrate RAG-based architectures' ability to revolutionize the way we conduct legal research by making complex data more easily understood and accessible. Future work could focus on developing standardized, multi-dimensional evaluation protocols that jointly assess correctness, faithfulness, relevance, clarity, and verifiability. Such protocols could incorporate human-in-the-loop evaluation, where legal experts validate system outputs alongside automated citation- and reasoning-based metrics, enabling more reliable and reproducible assessment across different legal AI systems.

**Figure 8:**
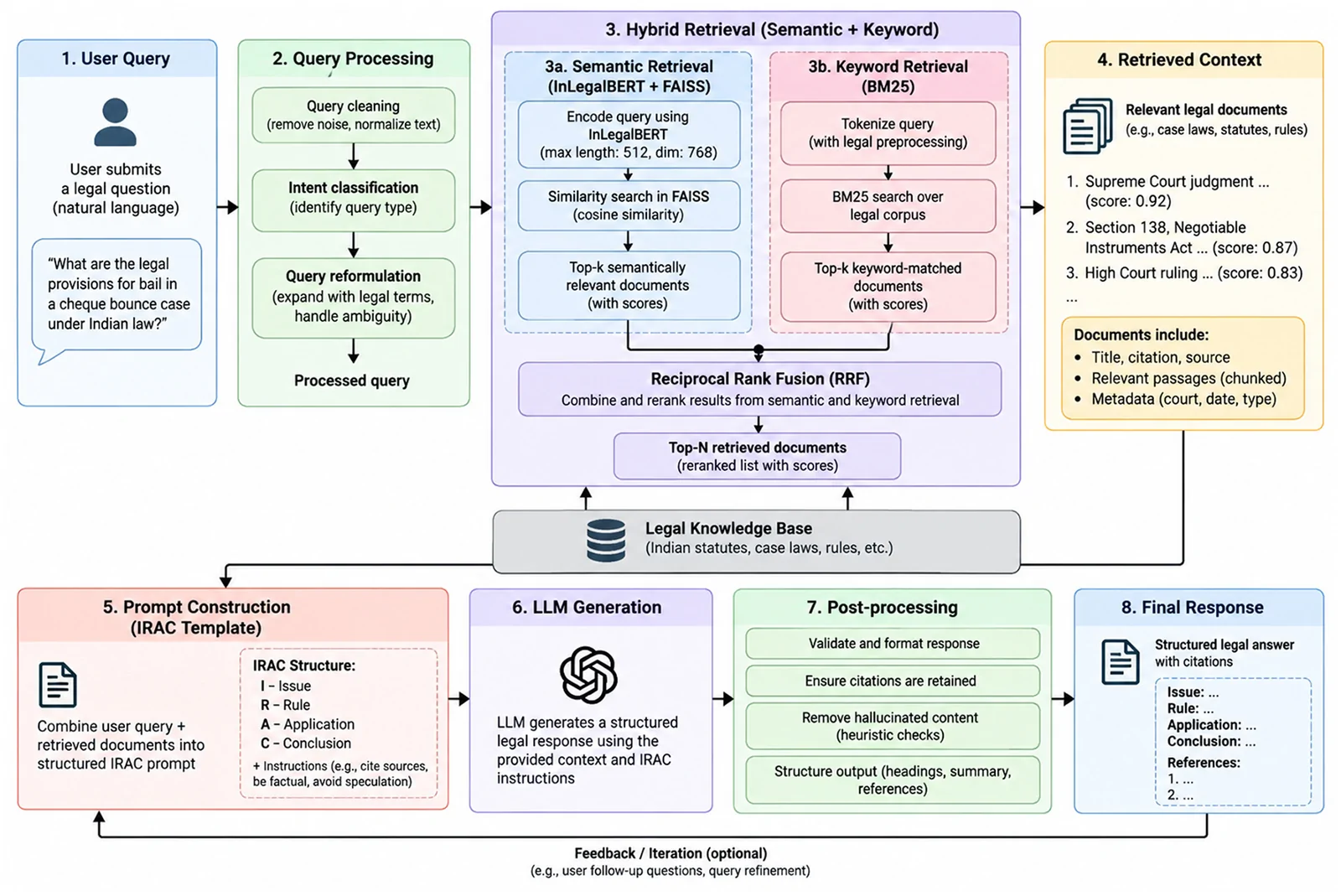
System flow diagram.

Several avenues exist for improving the proposed system in future work. One key direction is the development of advanced re-ranking mechanisms, such as cross-encoders, to enhance the precision of retrieved contexts by performing deeper query-document interaction before passing results to the language model. Additionally, incorporating hallucination detection and confidence scoring can significantly improve reliability by ensuring that generated responses are well grounded in retrieved evidence, thereby increasing trustworthiness in legal applications. As the framework primarily focuses on the Indian legal domain, its performance and applicability may be constrained when applied to other jurisdictions with different legal structures, terminology, and interpretative principles. The system is also dependent on the underlying legal corpus and language models, which may contain biases or incomplete representations of specific legal perspectives. Furthermore, the current implementation primarily supports English-language legal documents, limiting its applicability in multilingual legal environments. The system may also face challenges in interpreting highly nuanced legal arguments that require extensive contextual, jurisdiction-specific, or domain expertise. Future work can address these limitations by incorporating multilingual legal models and corpora, developing adaptable retrieval mechanisms for different jurisdictions, and enhancing legal reasoning capabilities to better capture complex legal nuances and contextual interpretations. Future research should also consider the societal and ethical implications of trustworthy explanations in legal AI systems. Although transparent, evidence-grounded explanations can improve user confidence and accessibility, they may also create risks of misinterpretation or over trust, particularly when users perceive AI-generated explanations as authoritative legal advice. Therefore, future systems should incorporate appropriate uncertainty communication, source verification, and human oversight to ensure that explanations support informed decision-making without encouraging excessive reliance on automated outputs. Finally, the inclusion of expert evaluation and user feedback mechanisms can support continuous system improvement, ensuring better alignment with real-world legal practices and enhancing both retrieval and generation quality over time.

## Data Availability

Publicly available datasets were analyzed in this study. This data can be found here: https://data.mendeley.com/datasets/gf8n8cnmvc/2Zenodo(2022) and https://zenodo.org/records/7151679.
